# Successful coronary sinus reducer implantation in a patient with a pre-existing left ventricular pacing lead: a case report

**DOI:** 10.1093/ehjcr/ytag356

**Published:** 2026-05-14

**Authors:** Marco Ciardetti, Michele Coceani, Augusto Esposito, Alberto Aimo, Cataldo Palmieri

**Affiliations:** Fondazione Toscana Gabriele Monasterio, Piazza Martiri della Libertà 33, Pisa and Massa 56124, Italy; Fondazione Toscana Gabriele Monasterio, Piazza Martiri della Libertà 33, Pisa and Massa 56124, Italy; Fondazione Toscana Gabriele Monasterio, Piazza Martiri della Libertà 33, Pisa and Massa 56124, Italy; Fondazione Toscana Gabriele Monasterio, Piazza Martiri della Libertà 33, Pisa and Massa 56124, Italy; Interdisciplinary Center for Health Sciences, Scuola Superiore Sant’Anna, Piazza Martiri della Libertà 33, Pisa 56124, Italy; Fondazione Toscana Gabriele Monasterio, Piazza Martiri della Libertà 33, Pisa and Massa 56124, Italy

**Keywords:** Reducer, Coronary sinus, Refractory angina, Cathether, Lead, Case report

## Abstract

**Background:**

Coronary sinus reducer (CSR) implantation offers symptomatic relief in patients with refractory angina who remain symptomatic despite optimal medical therapy and repeated revascularization procedures. Pre-existing coronary sinus pacing leads can complicate device delivery and raise concerns about lead displacement or venous injury.

**Case summary:**

A 77-year-old man with ischaemic heart failure (left ventricular ejection fraction 33%), a cardiac resynchronization therapy defibrillator (CRT-D; upgraded from a previously implanted implantable cardioverter-defibrillator), and Canadian Cardiovascular Society Class III refractory angina after extensive revascularization was evaluated for alternative treatment. Despite multiple interventions addressing recurrent in-stent restenoses, disabling angina persisted, prompting referral for CSR implantation. Right internal jugular venous access was selected for favourable coronary sinus alignment, with ultrasound guidance and use of an extrastiff guidewire and selective venography to delineate anatomy. Advancement of the 12 Fr delivery sheath was impeded by a prominent Thebesian valve; a 5 mm balloon was inflated at the obstruction and then partially inflated to facilitate ‘balloon-assisted tracking,’ enabling safe sheath passage without injuring the venous wall or disturbing the left ventricular lead. The CSR was deployed in the mid-distal coronary sinus with stable pacing parameters and no immediate procedural complications. During follow-up the patient remained free of angina.

**Discussion:**

This case demonstrates technical feasibility and safety of CSR implantation in the setting of a pre-existing left ventricular pacing lead and challenging venous anatomy when meticulous access selection, balloon-assisted tracking, angiographic confirmation, and systematic monitoring of lead electrical parameters are employed.

Learning points

- Coronary sinus reducer implantation can be safely performed despite a pre-existing left ventricular pacing lead with meticulous coronary sinus cannulation and intraprocedural lead assessment.
- Balloon-assisted tracking from a right internal jugular approach can help overcome a prominent Thebesian valve during coronary sinus reducer delivery.
- The coronary sinus reducer provides a minimally invasive treatment option for refractory angina in patients unsuitable for further revascularization.

## Introduction

Coronary sinus reducer (CSR) implantation is a promising therapeutic strategy for patients with refractory angina pectoris.^1^ This technique involves the placement of an hourglass-shaped device within the coronary sinus (CS), redistributing myocardial blood flow to ischaemic areas.^2,3^ Pre-existing devices and anatomical abnormalities cause a risk of mechanical interference between devices and lead dislodgement.^3,4^ Although feasibility in device-bearing patients has been reported,^4–8^ evidence on CSR implantation in the presence of a pre-existing LV lead remains limited.

## Summary figure

**Figure:**
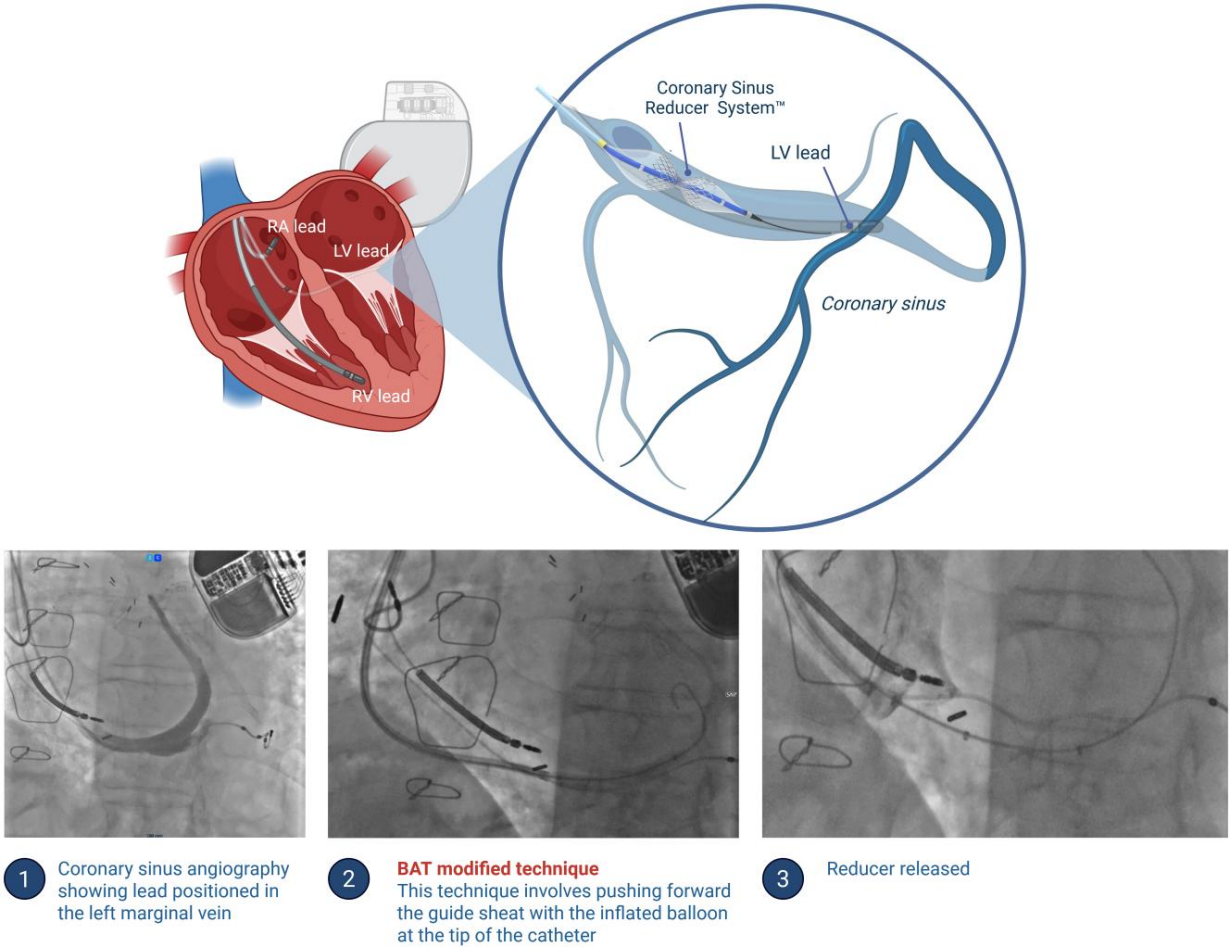
Coronary sinus reducer implantation in a patient with a pre-existing CRT-D left ventricular lead. The upper panel schematically illustrates the relationship between the coronary sinus reducer system, the coronary sinus, and the pre-existing left ventricular (LV) lead positioned in a left marginal vein in a patient with cardiac resynchronization therapy. The lower panels show the main procedural steps: (i) coronary sinus angiography confirming the anatomy and the course of the LV lead within the left marginal vein; (ii) modified balloon-assisted tracking (BAT) technique used to facilitate advancement of the guide sheath across the obstructed coronary sinus ostium/Thebesian valve by pushing the sheath over a partially inflated balloon; and (iii) final release of the coronary sinus reducer in the mid-distal coronary sinus, with preservation of LV lead position.

## Case presentation

A 77-year-old man with ischaemic heart failure presented with refractory angina [Canadian Cardiovascular Society (CCS) Class III] despite multiple previous coronary revascularization procedures and optimal medical therapy including metoprolol 100 mg b.i.d., isosorbide mononitrate extended release 120 mg daily, and ranolazine 750 mg b.i.d. His medical history included Type 2 diabetes, hypertension, chronic kidney disease, and former smoking. He underwent coronary artery bypass grafting in 1990 with grafts placed from the saphenous vein to the right coronary artery and obtuse marginal branch (OM) and a left internal mammary artery to the left anterior descending artery. During the following years, he underwent multiple coronary revascularization procedures involving the circumflex (CX)/OM territory and posterolateral branches because of graft failure and recurrent in-stent restenosis. Because of persistent left ventricular systolic dysfunction [left ventricular ejection fraction (LVEF) 33%], a single-chamber cardioverter-defibrillator was implanted in 2015 for primary prevention and later upgraded to cardiac resynchronization therapy with defibrillator (CRT-D) in 2017. Despite further revascularization procedures, he remained highly symptomatic. A coronary angiography confirmed advanced coronary artery disease with no further revascularization options.

In September 2024, the patient underwent CSR implantation. Right internal jugular vein access was chosen due to the favourable anatomic alignment with the CS (see Supplementary material online, *Video S1*). Ultrasound guidance minimized the risk of vascular complications. After puncture, a 0.035-inch extrastiff guidewire was inserted to provide adequate support for larger catheters. A 6 Fr multipurpose catheter was advanced to engage the coronary sinus ostium and perform selective venography, allowing visualization of venous structures and delineation of any valvular or anatomical variations within the CS. Once the CS ostium was engaged, the 12 Fr guide catheter for the CSR system was introduced. A prominent Thebesian valve prevented the sheath from advancing distally (see Supplementary material online, *Video S2*). Forceful sheath advancement could have increased the risk of CS dissection or perforation, with possible pericardial effusion/tamponade, as well as LV lead dislodgement or structural damage, potentially resulting in loss of biventricular pacing and need for lead revision. A 5 mm peripheral balloon was then threaded over the guidewire and inflated at the site of resistance to dilate or fracture the valve. Subsequently, the partially inflated balloon tip was used as a guide for the sheath (‘balloon-assisted tracking’). By gently advancing the deflated sheath over the partially inflated balloon, valvular obstruction was overcome without injuring the CS wall and minimizing mechanical interaction with the LV lead. After the 12 Fr delivery sheath crossed the valve, the CSR system was passed over the wire and positioned in the mid-distal CS (see Supplementary material online, *Video S3*). Proper alignment ensured that the device remained stable without compressing or dislodging the LV lead, which lay in a left marginal branch of the CS. Deployment involved unsheathing the CSR and allowing it to self-expand against the venous wall (see Supplementary material online, *Videos S4* and *S5*). Repeat angiography confirmed correct placement and vessel patency (see Supplementary material online, *Video S6*). Pacing lead measurements were obtained before and after CSR deployment to confirm stable electrical parameters.

After CSR implantation, the patient was started on dual antiplatelet therapy. A bleeding gastro-enteric anastomotic ulcer was managed conservatively with proton pump inhibitors and transfusions. Following atrial flutter detection, in November 2024 dual antiplatelet therapy was replaced with edoxaban, later stopped due to recurrent bleeding. Given the high thromboembolic and bleeding risk, left atrial appendage occlusion was performed in December 2024. As of April 2026, the patient is free from angina (CCS I), syncope, or palpitations, with NYHA Class II dyspnoea. Therapy is unchanged, and LV systolic function has improved slightly to 35%.

## Discussion

We report successful CSR implantation in a patient with ischaemic heart failure, CRT-D, and extensive prior revascularization. The pre-existing CS pacing lead increased procedural complexity. Feasibility in such cases depends largely on venous anatomy: an LV lead in a side branch that preserves an adequate main CS segment facilitates reducer delivery, whereas a lead near the intended landing zone may hinder catheter advancement and increase the risk of lead displacement or damage. In this patient, the LV lead was positioned in a left marginal branch, allowing safe deployment of the CSR in the mid-distal CS without apparent conflict. These findings suggest that, in device-bearing patients, procedural success depends more on lead position and CS anatomy than on the mere presence of an LV lead.

Only few comparable reports are available. Tsiachris *et al*.^4^ described the first parallel implantation of a CSR and a biventricular pacemaker, demonstrating that simultaneous coexistence of the two systems within the coronary venous circulation is technically possible. Farooqui *et al*.^7^ reported successful CSR implantation in a patient with a pre-existing LV lead, using a right internal jugular approach, and highlighted the potential long-term issue that trapping the lead between the reducer and the CS wall may complicate future extraction. Di Lorenzo *et al*.^8^ described CSR implantation in a patient with CRT-D, again underscoring that lead damage or dislodgement is a central procedural concern. Compared with these reports, the distinctive feature of the present case is that, in addition to the pre-existing LV lead, a prominent Thebesian valve created a second mechanical obstacle to sheath advancement, requiring a specifically adapted delivery strategy.

Potential pitfalls extend beyond difficulty in device delivery and include increased risk of CS injury during catheter or sheath manipulation. In addition, interaction between the delivery system, the reducer, and the LV lead may cause lead dislodgement or damage, with potential loss of capture, ineffective resynchronization, worsening heart failure symptoms, or the need for repeat device intervention. For these reasons, the procedure should be approached with careful venographic assessment, gentle catheter manipulation, and systematic checking of lead function before and after device deployment.

**Figure 1:**
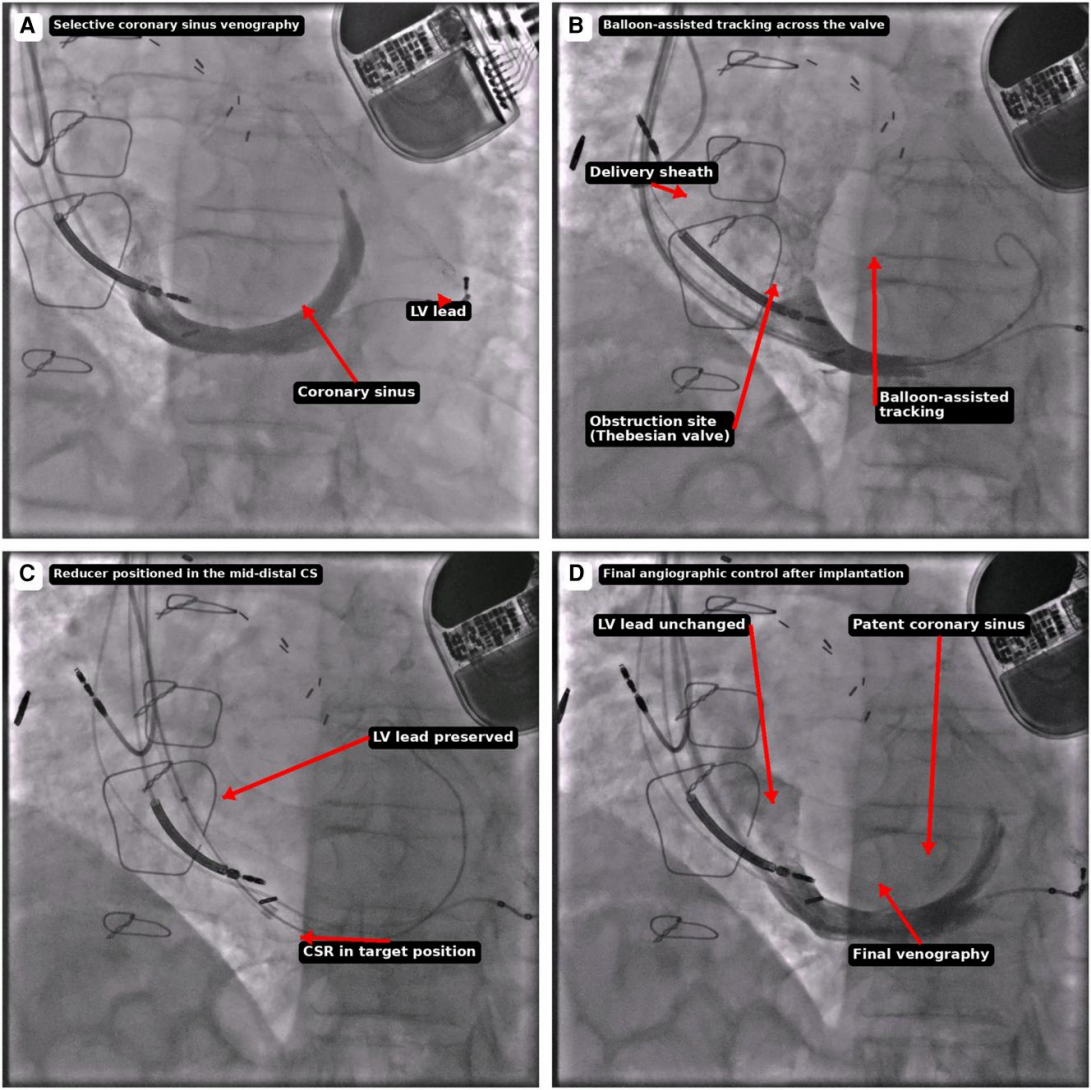
Key procedural steps of coronary sinus reducer implantation in a patient with a pre-existing left ventricular pacing lead. (*A*) Selective coronary sinus venography showing the coronary sinus anatomy and the course of the left ventricular pacing lead. (*B*) Balloon-assisted tracking used to overcome resistance at the level of a prominent Thebesian valve and facilitate advancement of the delivery sheath. (*C*) Coronary sinus reducer positioned in the mid-distal coronary sinus with preserved spatial relationship to the pre-existing left ventricular lead. (*D*) Final angiographic control after deployment, confirming correct device position, preserved coronary sinus patency, and stable left ventricular lead position without evidence of dissection, perforation, or contrast extravasation.

Balloon-assisted tracking involves partial inflation of a balloon catheter at the obstruction site, providing stability and allowing controlled advancement of the delivery sheath^9^ (*Figure 1* and *Summary Figure*). In this case, this strategy was particularly useful because it reduced the need for forceful manipulation across the Thebesian valve, potentially lowering the risk of both venous injury and lead-related complications. This technical nuance also differentiates the present report from previously published cases, which primarily focused on lead–reducer coexistence but did not specifically address balloon-assisted sheath tracking to overcome valvular obstruction at the CS ostium.^4,7,8^

The choice of the right internal jugular access was important because it provided a more favourable alignment with the CS and facilitated controlled catheter engagement. Balloon-assisted tracking was particularly helpful in overcoming the Thebesian valve while reducing the need for forceful advancement of the 12 Fr sheath. This manoeuvre likely decreased the risk of both venous injury and traction on the pre-existing LV lead. In addition, intraprocedural fluoroscopic surveillance, repeat venography, and post-deployment assessment of pacing parameters were essential to confirm the absence of CS injury and to document preserved lead integrity and electrical performance.

Careful intraprocedural monitoring and angiographic confirmation ensure optimal CSR positioning without compromising existing pacing leads. Electrical parameter assessments of pacing leads before and after CSR deployment are essential to verify lead integrity and stability. An additional aspect that deserves consideration is the bailout strategy in the event of LV lead failure. Had lead dislodgement or dysfunction occurred, immediate reassessment would have been required to determine whether lead repositioning or revision was feasible. The reverse scenario has already been described: Bontempi *et al*.^10^ first reported LV lead implantation after prior CSR placement, and later reports by Grebmer *et al*.^5^ and Costantino *et al*.^6^ showed that CRT lead implantation, extraction, or re-implantation may remain feasible after CSR, either by crossing the reducer or by using a suitable proximal tributary under favourable anatomical conditions. These observations are relevant for the present case because they suggest that future lead revision might still be possible if required, although clearly at the cost of greater technical complexity.

In conclusion, this case demonstrates that CSR implantation can be safely and effectively performed in patients with refractory angina, a CRT-D system, and complex CS anatomy. This outcome underscores the role of CSR as an important adjunctive therapy in carefully selected cases with no further revascularization options and refractory ischaemic symptoms. Careful assessment of the coronary venous anatomy is crucial to determine feasibility and to anticipate technical challenges. When lead position is favourable but ostial or valvular anatomy impedes sheath progression, a tailored strategy combining right internal jugular access with balloon-assisted tracking may allow safe CSR delivery while preserving lead integrity. As more clinical experience is gathered, future studies should focus on defining standardized protocols and refining techniques for patients with device-related anatomical complexities.

## Supplementary Material

ytag356_Supplementary_Data

## Data Availability

The data underlying this article will be shared on reasonable request to the corresponding author.

## Lead author biography

Dr. Alberto Aimo, MD, PhD, FESC, FHFA, is a cardiologist at the Fondazione Toscana Gabriele Monasterio, a division of the Italian National Research Council, and a researcher at the Scuola Superiore Sant’Anna in Pisa, Italy. His research primarily focuses on heart failure and cardiomyopathies, with an emphasis on the use of biomarkers and advanced imaging for diagnosis and risk stratification, as well as drug and device therapies and structural interventions in heart failure.

## Notes

**Consent:** The patient provided written informed consent to the presentation of his case in an anonymised form, in compliance with COPE guidelines.
